# Combining pulmonary endarterectomy and balloon pulmonary angioplasty in chronic thromboembolic pulmonary hypertension: a narrative review of hybrid therapy approaches

**DOI:** 10.1183/23120541.00414-2025

**Published:** 2025-12-15

**Authors:** Hiroyuki Fujii, Yuichi Tamura, Sarasa Isobe, Miki Sakamoto, Keiko Sumimoto, Kenichi Yanaka, Kenji Okada, Noriaki Emoto, Hiromasa Otake, Yu Taniguchi

**Affiliations:** 1 Pulmonary Hypertension Center, International University of Health and Welfare Mita Hospital, Tokyo, Japan; 2 Division of Cardiovascular Medicine, Department of Internal Medicine, Kobe University Graduate School of Medicine, Kobe, Japan; 3 Division of Cardiovascular Surgery, Department of Surgery, Kobe University Graduate School of Medicine, Kobe, Japan; 4 Laboratory of Clinical Pharmaceutical Science, Kobe Pharmaceutical University, Kobe, Japan

## Abstract

The management of chronic thromboembolic pulmonary hypertension (CTEPH) has changed dramatically over the past two decades, with improved prognosis owing to the availability of pulmonary endarterectomy (PEA), balloon pulmonary angioplasty (BPA) and approved medications. Recently, treatment strategies combining these options have been determined by a multidisciplinary team according to localisation, characteristics, haemodynamics and comorbidities. This review summarises recent advances in hybrid therapy, combining PEA and BPA as a treatment approach for CTEPH, and discusses future perspectives.

## Shareable abstract

**Hybrid therapy, which combines the strengths of PEA and BPA, is a promising approach to enhance patient outcomes in CTEPH. Continued advancements in CTEPH management are anticipated, emphasising the importance of individualised patient-centred care.** https://bit.ly/43t5Wsf

## Overview of the study.

**Figure:**
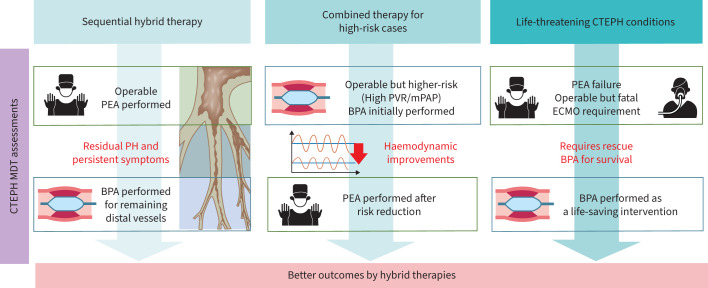
BPA: balloon pulmonary angioplasty; CTEPH: chronic thromboembolic pulmonary hypertension; ECMO: extracorporeal membrane oxygenation; MDT: multidisciplinary team; mPAP: mean pulmonary arterial pressure; PEA: pulmonary endarterectomy; PH: pulmonary hypertension; PVR: pulmonary vascular resistance.

**Lessons for clinicians**

Recent advancements suggest that combining PEA and BPA in a hybrid therapy approach may improve outcomes in patients with CTEPH.

A multidisciplinary approach should be considered, including hybrid therapy, to tailor the optimal treatment strategies for each individual.

## Introduction

Chronic thromboembolic pulmonary hypertension (CTEPH) is characterised by stenosis and obstruction of the pulmonary arteries due to nonresolving organised thromboemboli, leading to increased pulmonary vascular resistance (PVR), pulmonary hypertension (PH) and right heart failure. If left untreated, the prognosis of patients with CTEPH is very poor, with a 5-year survival rate of 10% in patients with a mean pulmonary artery pressure (mPAP) of >50 mmHg [1]. Pulmonary endarterectomy (PEA) is the standard treatment for operable CTEPH. However, a significant number of patients are deemed inoperable, with 13% in the United States [2] and 36.6% in Europe and Canada [3], according to the registers. Balloon pulmonary angioplasty (BPA) has emerged as an endovascular procedure for widening narrowed or obstructed pulmonary arteries. Its efficacy was first reported in 2001 [4]. Since then, many studies have demonstrated favourable clinical outcomes and safety profiles for BPA, thus establishing BPA as a treatment option for inoperable CTEPH [5]. Currently, three options are available for treating CTEPH, namely PEA, BPA and medication. Recently, the combinations of these options have been proposed at the 7th World Symposium on Pulmonary Hypertension [6]. This review aims to provide an overview of recent advances in hybrid therapy combining PEA and BPA as a treatment approach for CTEPH and discuss future perspectives.

## Challenge of residual organised thromboemboli after PEA

PEA is the gold-standard treatment for CTEPH because it can be used directly to remove organised thromboemboli from pulmonary arteries. In 1993, Jamieson *et al*. [7] reported good outcomes and safety profiles in 323 patients who underwent PEA. Subsequently, the mortality rate decreased from 17% in the first 200 patients to 4.4% in 2002 [8]. More recently, Madani *et al*. [9], from the same institution, reported patients with pre-operative PVR of more than 1000 dyn·s·cm^−5^ had an overall mortality of 4.1% (down from 9.3%) compared with 1.6% for patients with PVR of less than 1000 dyn·s·cm^−5^ (down from 2.0%), indicating the improved safety of the procedure. Similarly, large European centres have reported excellent haemodynamic improvements and safety, with an overall perioperative mortality rate of 4.7% [10].

However, residual PH due to peripheral chronic thromboemboli that are inaccessible by PEA remains a significant problem. The rate of residual PH after PEA is reportedly 11.4% in a single centre in Japan [11], 31% in the Netherlands [12], 31% and 35% in multicentre studies in the UK [13, 14], and 16.7% in other European and Canadian centres [10]. Moreover, Hsieh *et al*. [15] reported that in a recent meta-analysis of 25 studies involving 4686 patients, 25% of patients had residual PH. In this report, the definition of residual PH was determined using a cut-off value of mPAP ≥25 mmHg in most of the 12 studies, mPAP ≥30 mmHg in two studies, mPAP ≥25 mmHg and PVR ≥3.75 Wood units in one study, and mPAP ≥25 mmHg and PVR ≥240 dyn·s·cm^−5^ in another study. Cannon *et al*. [16] demonstrated that patients with residual mPAP ≥38 mmHg and PVR ≥425 dyn·s·cm^−5^ after PEA correlated with worse long-term survival. In other large PEA cohorts, most patients who died after PEA had residual PH [8, 17]. After successful PEA, some patients are found to have PH subsequently despite not having PH at the initial assessment [16]. Therefore, regular follow-up is necessary to detect recurrent PH after PEA.

How to treat such residual or recurrent PH after PEA remains a critical challenge. The aetiology of persistent PH after PEA is attributed to pulmonary microvascular disease (PMD), the persistence of endoluminal fibrotic material, which could not be removed by PEA, and recurrent thromboembolic events [18]. To improve PMD after PEA, pulmonary vasodilators approved for pulmonary arterial hypertension are effective. Riociguat in patients with persistent or recurrent PH after PEA was associated with improvement in the 6-min walk distance in the CHEST-1 study [19] and its long-term benefits in the CHEST-2 study [20]. As a result of these studies, riociguat is the first drug approved for residual PH after PEA. Although a promising therapy, riociguat does not remove the obstructive lesions and its efficacy is limited in cases with a large amount of residual endoluminal fibrotic material. To improve endoluminal fibrotic material after PEA, additional revascularisation should be considered. However, additional PEA is often not considered effective because of the high risks associated with repeated sternotomies and the difficulty in accessing peripheral thromboemboli. The efficacy and safety of adding BPA after PEA have recently been reported, offering a new therapeutic option.

## Hybrid therapy

Thus, hybrid therapy offers new possibilities for treating CTEPH. This section divides hybrid therapy into three main categories (see graphical abstract).

1) Sequential hybrid therapy: This strategy involves performing BPA after PEA to treat residual lesions that cannot be treated using PEA alone. It is effective for treating peripheral thromboembolic lesions that are inaccessible during surgery.
2) Combined therapy for high-risk cases: A strategic combination of BPA and PEA is used to reduce the surgical risk in patients with severe haemodynamic compromise who are at high surgical risk. BPA is performed before PEA for improved haemodynamics.
3) Rescue BPA for life-threatening PH: In cases where PEA has failed or life-threatening PH persists despite other therapies, BPA is used as a rescue treatment option.

These three treatment strategies are often effective, and the indications are determined by a multidisciplinary team (MDT) at experienced centres. This section presents evidence for each of these strategies and discusses hybrid CTEPH treatment approaches by presenting representative cases from our experiences.

### 1) Sequential hybrid therapy

BPA has emerged as an effective treatment for patients with residual PH after PEA. In 2015, Shimura *et al*. [21] reported that performing additional BPA in nine patients with CTEPH who had residual PH after PEA significantly improved PVR from a median of 8.1 (interquartile range 6.1–12.3) Wood units to 4.2 (interquartile range 2.8–4.8) Wood units. They reported one case of vascular injury with haemoptysis (0.6% of treated vessels) and one case of reperfusion pulmonary oedema (2.3% of 44 sessions).

Subsequently, studies from various countries by Yanaka *et al*. [22], Araszkiewicz *et al*. [23], Ito *et al*. [24] and Kirkby *et al*. [25] have reported the efficacy of additional BPA after PEA, all demonstrating improved haemodynamics. However, Kirkby *et al*. [25] reported that haemodynamic improvement was less pronounced than that in primary BPA for inoperable patients. The appropriate timing for additional BPA after PEA is unknown. Previous studies from Japan showed that additional BPA performed 4.1–7.3 months after PEA could improve haemodynamic of patients with residual or recurrent PH [21, 22]. Complications requiring treatment were observed in 2–16% of cases and no serious complications requiring extracorporeal membrane oxygenation (ECMO), endotracheal intubation or death were reported (supplementary file S1).

The main concern with the use of additional BPA after PEA is the potential fragility of the vessel walls following surgery. However, these studies indicate that BPA can be safely performed as early as 4 months after PEA, with risks comparable to those of regular BPA. This finding suggests that BPA is a safe and effective treatment for patients who continue to exhibit PH during follow-up after PEA.

Furthermore, some patients continue to have limited exercise capacity after PEA despite the normalisation of resting PAP and PVR [26]. Bonderman *et al*. [27] reported that patients with persistent exertional dyspnoea display an abnormal pulmonary haemodynamics response to exercise, characterised by increased PVR and decreased pulmonary arterial compliance after successful PEA. This study suggests some patients after PEA remain with exercise-induced PH and decreased ventilatory efficiency. Inami *et al*. [28] reported that BPA for chronic thromboembolic pulmonary disease (CTEPD) without PH can improve exercise capacity and ventilatory efficiency. In this study, exercise right heart catheterisation (RHC) showed a reduction in mPAP/cardiac output (CO) slope and cardiopulmonary exercise tests showed a reduction in minute ventilation (*V*′_E_) *versus* carbon dioxide production (*V*′_CO_2__) slope at follow-up, respectively. To evaluate patients who remain symptomatic after PEA, it is useful to assess parameters such as *V*′_E_ *versus V*′_CO_2__ slope in cardiopulmonary tests, which reflects exercise ventilatory efficiency or mPAP/CO slope in exercise RHC. Therefore, if these parameters are not normal, BPA might be considered even in CTEPD without PH. Patients who have undergone PEA without resting PH but with residual symptoms may also have these abnormal parameters. However, there is insufficient evidence to support BPA efficacy in these patients and this remains a future challenge. With an increasing number of reports confirming the efficacy and safety of additional BPA after PEA, it is anticipated that more cases will be considered for this indication at PH centres in the future.

#### Case 1 (figure 1)

A 61-year-old woman presented with shortness of breath on exertion and oedema for the previous 3 months. The patient had no significant medical history. Physical examination revealed signs of right heart failure. Diagnostic workup, including RHC, showed moderate PH (mPAP 38 mmHg), reduced CO (2.05 L·min^−1^) and elevated PVR (858 dyn·s·cm^−5^). Her symptoms were classified as World Health Organisation Functional Class (WHO-FC) III. She was evaluated by an MDT and was considered a suitable candidate for PEA. PEA was successfully performed without complications. However, after PEA, the patient still experienced symptoms that were classified as WHO-FC II. Follow-up RHC showed residual PH (mPAP 30 mmHg, PVR 463 dyn·s·cm^−5^) and pulmonary angiography revealed residual peripheral lesions. The MDT discussed the case and decided to perform additional BPA to address the residual lesions. After completing the four BPA sessions, her symptoms improved significantly to WHO-FC I. Follow-up RHC demonstrated normalised haemodynamics (mPAP 15 mmHg, PVR 193 dyn·s·cm^−5^), indicating a successful outcome of the sequential hybrid therapy combining PEA and BPA.

**FIGURE 1:**
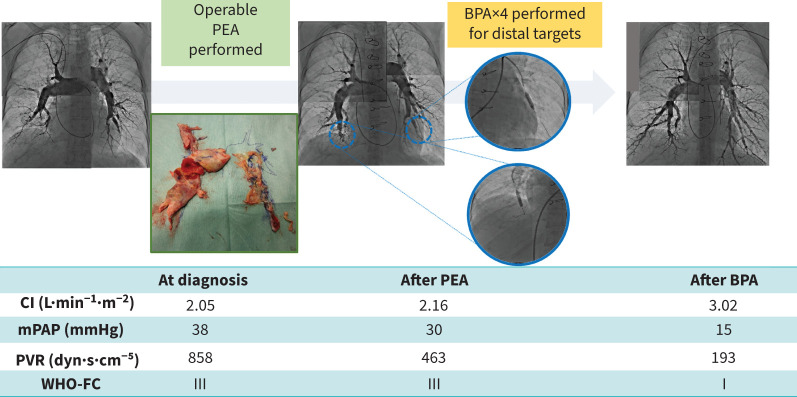
Clinical course of case 1. BPA: balloon pulmonary angioplasty; CI: cardiac index; mPAP: mean pulmonary arterial pressure; PEA: pulmonary endarterectomy; PVR: pulmonary vascular resistance; WHO-FC: World Health Organization functional class.

### 2) Combined therapy for high-risk cases

PEA is associated with a high surgical risk in patients with CTEPH and severe haemodynamic compromise. Madani *et al*. [9] reported that the mortality rate of PEA was 1.6% in patients with pre-operative PVR <1000 dyn·s·cm^−5^; however, it increased to 4.1% in those with PVR >1000 dyn·s·cm^−5^. Similarly, Jamieson *et al*. [8] found that 18 out of 22 patients who died after PEA had a pre-operative PVR >1000 dyn·s·cm^−5^. Thus, high pre-operative PVR is a significant challenge for PEA.

To address this issue, pre-operative BPA has been explored as a means of reducing surgical risks. Shimahara *et al*. [29] compared 21 patients with high-risk CTEPH who underwent BPA before PEA with 37 patients who underwent direct PEA. They reported that although four of the 21 patients (19.1%) experienced complications requiring noninvasive positive pressure ventilation (NPPV), the group receiving BPA before PEA had a significantly lower rate of composite events than the direct PEA group (4.8% *versus* 35.1%, p=0.011). Additionally, BPA before PEA significantly decreased mPAP and PVR and increased CO. Concerns have been raised that BPA performed before PEA may cause pulmonary artery dissection, making surgical access difficult and increasing the risk of vascular injury. However, Shimahara *et al*. [29] reported no such events, suggesting that pre-operative BPA can be safely incorporated into the treatment plan for operable but haemodynamically high-risk patients.

Wiedenroth *et al*. [30] introduced a completely new approach combining PEA and BPA during the same procedure for high-risk cases and pulmonary artery obstruction that is only partially accessible for surgery. This hybrid approach was attempted because PEA alone was predicted to increase the risk of post-operative right heart failure and death without adequately reducing RV afterload. This is one of the “combined therapy for high-risk cases” that strategically combines PEA and BPA. Such an approach would be discussed by an experienced MDT, depending on the experience of the centre for rare cases with unilateral obstruction operable and contralateral inoperable.

#### Case 2 (figure 2)

A 54-year-old woman was diagnosed with CTEPH after experiencing shortness of breath for 6 months. RHC revealed severe PH with an mPAP of 52 mmHg and PVR of 1126 dyn·s·cm^−5^. Pulmonary angiography revealed central lesions in the right pulmonary artery and peripheral lesions on the left side. Due to the high surgical risk, BPA was performed before PEA. During the BPA session, the patient experienced haemoptysis requiring NPPV; however, her condition quickly improved and the mPAP and PVR decreased to 42 mmHg and 891 dyn·s·cm^−5^, respectively. One and a half months after the BPA, PEA was performed without complications. Haemodynamics improved significantly to an mPAP of 16 mmHg and PVR of 219 dyn·s·cm^−5^, and the patient's symptoms improved to WHO-FC I.

**FIGURE 2:**
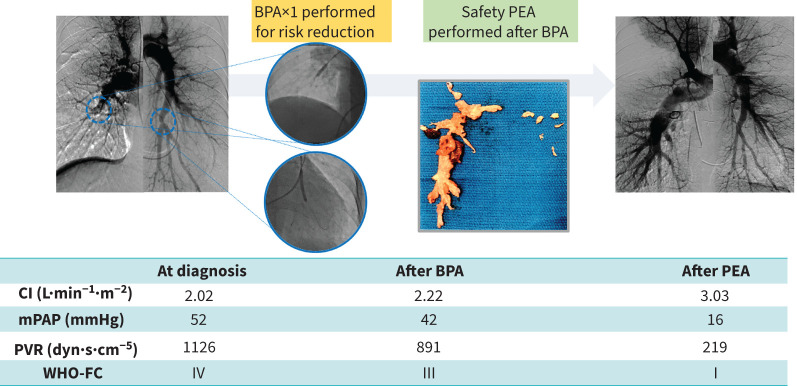
Clinical course of case 2. BPA: balloon pulmonary angioplasty; CI: cardiac index; mPAP: mean pulmonary arterial pressure; PEA: pulmonary endarterectomy; PVR: pulmonary vascular resistance; WHO-FC: World Health Organization functional class.

### 3) Rescue BPA for life-threatening CTEPH

The management of patients with CTEPH who experience rapidly progressive PH and require ECMO support is particularly challenging. Traditional treatments may not be sufficient for critically ill patients. Recent case reports have explored the use of rescue BPA as a life-saving intervention.

Sumimoto *et al*. [31] described two cases of rescue BPA in patients with acute pulmonary embolism (PE) superimposed on CTEPH who experienced rapidly progressing PH and required ECMO support. DiChiacchio *et al.* [32] reported that 35.5% of patients who underwent acute surgical pulmonary embolectomy also had chronic thromboembolic disease confirmed by intraoperative endarterectomy; however, it is often unrecognised. Patients with PE who have rapidly progressive symptoms and higher systolic PAP should be considered for the possibility of underlying CTEPH [33]. Acute PE superimposed on CTEPH sometimes progresses rapidly, leading to rapid deterioration of right heart function and rescue BPA may be considered as an option. However, Sumimoto *et al*. [31] also reported that in one of these cases, severe alveolar haemorrhage occurred despite careful use of the technique, highlighting the risks involved. Nakamura *et al*. [34] performed rescue BPA in a patient with peripheral CTEPH who did not respond to PEA and required ECMO support and reported a favourable outcome. Conversely, Collaud *et al*. [35] reported three cases of rescue BPA under ECMO support after PEA failure, with two patients dying from multiple organ failure. These mixed outcomes indicate that while rescue BPA may be the only life-saving option in certain cases, its efficacy and safety in critically ill patients on ECMO have not yet been fully established. Severe haemodynamic compromise is a known risk factor for complications during BPA, including vascular injury and reperfusion pulmonary oedema. Therefore, rescue BPA should be cautiously considered and performed in high-volume centres with expertise in CTEPH treatment.

#### Case 3 (figure 3)

A 50-year-old woman was diagnosed with acute PE 2 years prior and was on anticoagulant therapy. She was also diagnosed with systemic lupus erythematosus following pleurisy 1 year prior and treated with corticosteroids (prednisone 60 mg·day^−1^). The symptoms had subsided and the corticosteroid dose was gradually reduced to prednisone 5 mg·day^−1^. She was admitted to the hospital with progressive exertional dyspnoea in the past few months. On admission, RHC revealed severe PH, mPAP 48 mmHg and PVR 920 dyn·s·cm^−5^. Her symptoms were classified as WHO-FC IV. Pulmonary angiography showed obstruction and stenosis from the proximal segmental branches. She was diagnosed with CTEPH and considered a suitable candidate for PEA by an MDT. PEA was performed, but due to the PH crisis, it was difficult to wean the patient from the cardiopulmonary pump and veno-arterial ECMO was started. She continued to have difficulty weaning off ECMO post-operatively with medication alone, so rescue BPA was performed on post-operative day 5. BPA was carefully performed using small balloons (2.0 and 3.0 mm in diameter) focusing on the right lower lobe artery. After the procedure, she experienced haemodynamic improvement immediately, allowing ECMO discontinuation 13 days after rescue BPA. She was discharged without assistance 3 months after hospitalisation. One additional BPA was performed later and her haemodynamics had been improved (mPAP 21 mmHg and PVR 308 dyn·s·cm^−5^). Her symptoms also improved to WHO-FC I.

**FIGURE 3:**
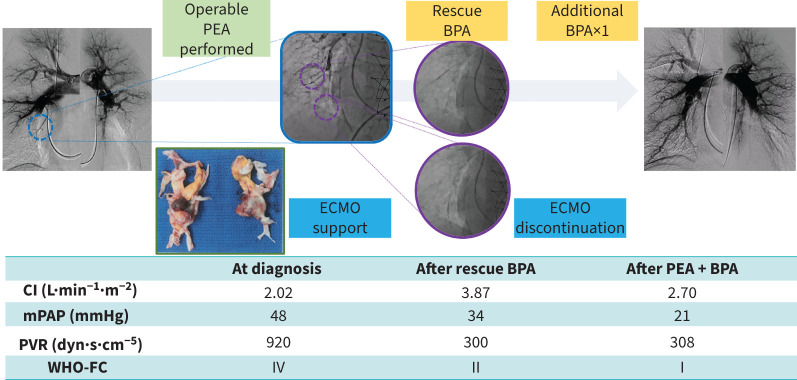
Clinical course of case 3. BPA: balloon pulmonary angioplasty; CI: cardiac index; mPAP: mean pulmonary arterial pressure; ECMO: extracorporeal membrane oxygenation; PEA: pulmonary endarterectomy; PVR: pulmonary vascular resistance; WHO-FC: World Health Organization functional class.

## Conclusion

The number of patients with CTEPH is expected to increase [36]. PEA has traditionally been the only treatment option for CTEPH. However, with the advent of additional options, such as BPA and medication, CTEPH is now treatable in almost all patients, offering a significantly improved prognosis. Given that haemodynamics, lesion localisation and comorbidities vary among patients, it is essential to consider a multidisciplinary approach, including hybrid therapy, to tailor the optimal treatment strategies for each individual. Hybrid therapy, which combines the strengths of PEA and BPA, is a promising approach to enhance patient outcomes. Continued advancements in CTEPH management are anticipated, emphasising the importance of individualised patient-centred care.

## Supplementary material

**Please note:** supplementary material is not edited by the Editorial Office, and is uploaded as it has been supplied by the author. Supplementary material 00414-2025.SUPPLEMENT
