# Supplementary material for: Combining pulmonary endarterectomy and balloon pulmonary angioplasty in chronic thromboembolic pulmonary hypertension: a narrative review of hybrid therapy approaches
Source: ERJ Open Res. 2025 Dec 15;11(6):00414-2025. doi: 10.1183/23120541.00414-2025 (PMC12704146; doi:10.1183/23120541.00414-2025)
Supplement: Supplementary file 1 [file 00414-2025.SUPPLEMENT.pdf]

Supplementary file 1. Summary of the findings of studies on the use of additional BPA after PEA, including the number of patients, timing after PEA, haemodynamic improvements, and complication rates

Authors	Year	Patients (n)	Period from PEA to 1st BPA (months)	PVR before BPA (dyne/sec/cm^{-5})	PVR after BPA (dyne/sec/cm^{-5})	ΔPVR	Severe haemoptysis*	Mechanical ventilation or ECMO
Shimura et al.	2015	9	4.1 (2.7 –7.9)	648 (488–984)	336 (224384)	-48 %	2.30 %	0 %
Yanaka et al.	2018	10	7.3 ± 2.3	386 ± 42	242 ± 39	-37 %	8.30 %	0 %
Araszkiewicz et al.	2019	15	28.1 ± 25.8	552 ± 184	348 ± 126	-37 %	2.80 %	0 %
Ito et al.	2021	25	unknown	392 ± 160	296 ± 40	-24 %	16.80 %	0 %
Kirkby et al.	2023	20	unknown	532 ± 172	454 ± 182	-15 %	unknown	unknown

Abbreviations: PEA, pulmonary endarterectomy; BPA, balloon pulmonary angioplasty; PVR, pulmonary vascular resistance; ECMO, extracorporeal membrane oxygenation

* Severe haemoptysis requiring embolisation, covered stents, or non-invasive positive pressure ventilation
